# DNA diet profiles with high‐resolution animal tracking data reveal levels of prey selection relative to habitat choice in a crepuscular insectivorous bird

**DOI:** 10.1002/ece3.6893

**Published:** 2020-10-16

**Authors:** Ruben Evens, Greg Conway, Kirsty Franklin, Ian Henderson, Jennifer Stockdale, Natalie Beenaerts, Karen Smeets, Thomas Neyens, Eddy Ulenaers, Tom Artois

**Affiliations:** ^1^ Max Planck Institute for Ornithology Eberhard‐Gwinner‐Straße Starnberg Germany; ^2^ Centre for Environmental Sciences Research Group: Zoology, Biodiversity and Toxicology Hasselt University Diepenbeek Belgium; ^3^ British Trust for Ornithology Thetford UK; ^4^ Cardiff School of Biosciences Cardiff UK; ^5^ Norwich Research Park University of East Anglia Norwich UK; ^6^ Faculty of Medicine & Health Sciences University of Nottingham Nottingham UK; ^7^ Agentschap Natuur en Bos Regio Noord‐Limburg Brussels Belgium

**Keywords:** DNA metabarcoding, food availability, foraging ecology, high‐throughput sequencing, lepidoptera

## Abstract

Given the global decline of many invertebrate food resources, it is fundamental to understand the dietary requirements of insectivores. We give new insights into the functional relationship between the spatial habitat use, food availability, and diet of a crepuscular aerial insectivore, the European Nightjar (*Caprimulgus europaeus*) by relating spatial use data with high‐throughput sequencing (HTS) combined with DNA metabarcoding. Our study supports the predictions that nightjars collect a substantial part of their daily nourishment from foraging locations, sometimes at considerable distance from nesting sites. Lepidopterans comprise 65% of nightjars' food source. Nightjars tend to select larger species of *Lepidoptera* (>19 mm) which suggests that nightjars optimize the efficiency of foraging trips by selecting the most energetically favorable—larger—prey items. We anticipate that our findings may shed additional light on the interactions between invertebrate communities and higher trophic levels, which is required to understand the repercussions of changing food resources on individual‐ and population‐level processes.

## Graphical abstract

We give new insights into the functional relationship between the spatial habitat use, food availability, and diet of a crepuscular aerial insectivore. Nightjars collect a substantial part of their daily nourishment from foraging locations which were presumed to be unsuitable for the species. These foraging habitats are found at considerable distances from breeding habitats and comprise grasslands and meadows.

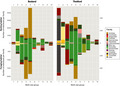

## 1 INTRODUCTION

The global decline of many insect communities (Sánchez‐Bayo & Wyckhuys, 2019; Saunders et al., 2020; Wagner, 2020) is negatively affecting higher trophic levels such as mammals, amphibians, and birds (Hallmann et al., 2017). Populations of avian aerial insectivores, such as swallows, swifts, and nightjars, are believed to be declining due to the loss of their prey (Ng et al., 2018; Smith et al., 2015). In most cases, the diet composition of these predatory species is unknown and it is still unclear when and where they collect their food within their summer home‐range. With the advent of modern tracking technologies and molecular techniques, we have the opportunity to combine information on the space use of such species (Evens et al., 2018; Ravache et al., 2020) and their specific diet (King et al., 2015; Pompanon et al., 2012).

Miniature tracking devices, such as radio telemetry and GPS‐tracking, now also allow us to reveal the intimate movement and habitat use of difficult‐to‐study species (Kays et al., 2015). In order to comprehend the spatial habitat use of species, it is fundamental to understand their dietary composition and food preference (Trevelline et al., 2016) and to investigate species' response to habitat loss or fragmentation (Meyer et al., 2008), particularly since many invertebrate taxa are in global decline (Saunders et al., 2020; Wagner, 2020). Similar to many other species, aerial insectivores can behave as central place foragers during the breeding season when vital, complementary resources, such as nesting sites and food, are distributed heterogeneously in time and space (Dunning et al., 1992; Evens et al., 2017; Kareiva & Odell, 1987; Michelot et al., 2017; Ripperger et al., 2015). Birds are assumed to balance the costs of traveling against the benefits of energy acquisition to maximize net‐energy gain (Hedenstrom & Alerstam, 1995; Wilson et al., 2012). When individuals live in fragmented landscapes, greater travel distances across unsuitable habitats may lead to increases in daily energy expenditure (Hinsley et al., 2008; Staggenborg et al., 2017) with direct implications for their survival probability (Panzacchi et al., 2009) and fecundity (Catry et al., 2013; Hinsley et al., 2008; Perrig et al., 2014; Ropert‐Coudert et al., 2004). Individuals may, therefore, select bigger, more profitable, prey to increase gross food intake (Emlen, 1966; Schoener, 1971; Stephens & Krebs, 1985).

To assess a species' dietary composition, traditional morphology‐based methods can be time consuming and often biased toward the identification of recognizable and intact undigested prey remains (Pompanon et al., 2012). Molecular techniques, such as high‐throughput sequencing (HTS) combined with DNA metabarcoding (Thompson & Newmaster, 2014), are increasingly utilized to assess the diet of predators (de Sousa et al., 2019) because they require no a priori information on prey composition (Pompanon et al., 2012; Valentini et al., 2009) and a wide range of prey can be identified to the genus‐ or species‐level (King et al., 2008). For this application, fecal samples are very useful as they contain residual prey DNA and can be collected with minimal disturbance to the focal species that would otherwise be difficult to approach or study (Pompanon et al., 2012).

Technological advances now provide an opportunity to investigate the linkages between individuals' spatial movements, habitat use, and diet, not previously possible for smaller terrestrial species (Carreiro et al., 2020; Groom et al., 2017). Investigating these linkages by combining multiple modern techniques will allow for deeper insights into the ecology of difficult‐to‐study species than would be possible from any one technique alone (Groom et al., 2017). One such a difficult‐to‐study species is the European Nightjar (*Caprimulgus europaeus*, hereafter referred to as nightjar), a crepuscular, aerial insectivorous bird. In Western Europe, nightjars mainly breed on open semi‐natural, low‐nutrient habitats, such as heathland, as well as open woodland (Conway et al., 2007; Evens et al., 2017). Nightjars have been assumed to forage predominantly around the nest vicinity (Sharps et al., 2015; Wichmann, 2004). However, it has been demonstrated that nightjars routinely forage in habitats presumed to be unsuitable for the species, such as extensively cultivated grasslands and wet grasslands (Alexander & Cresswell, 1990; Conway et al., 2019; Evens et al., 2017), which may often be many kilometers away from nest sites. Nightjars appear to select presumably food‐rich locations in order to optimize their net‐energy gain (Evens et al., 2018) and feed opportunistically, primarily on *Lepidoptera* followed by *Coleoptera* as well as *Diptera* and *Hymenoptera* (Sierro et al., 2001; Schlegel, 1967). Nevertheless, the exact contribution of these prey groups to the composition of nightjars' diet is unknown and it is unclear which component of their diet is collected at these foraging sites.

Here, we used a novel combination of high‐throughput sequencing (HTS) with DNA‐metabarcoding to assess the diet of adult nightjars, in relation to their spatial habitat use and food availability, in two Western‐European populations. To achieve this, we (a) screened fecal samples to identify prey‐DNA using C01 metabarcoding primers, (b) identified nesting and foraging sites using radio telemetry and GPS technology, and (c) measured food availability in nesting and foraging habitats. We predicted that (a) species of *Lepidoptera* comprise the main component of nightjars' diet, that (b) prey items could be related directly to foraging habitats, and (c) that prey consumption would be optimized to maximize efficiency that is, consumption of large items requiring least handing time for maximum profitability.

## 2 MATERIALS AND METHODS

**Figure 1:**
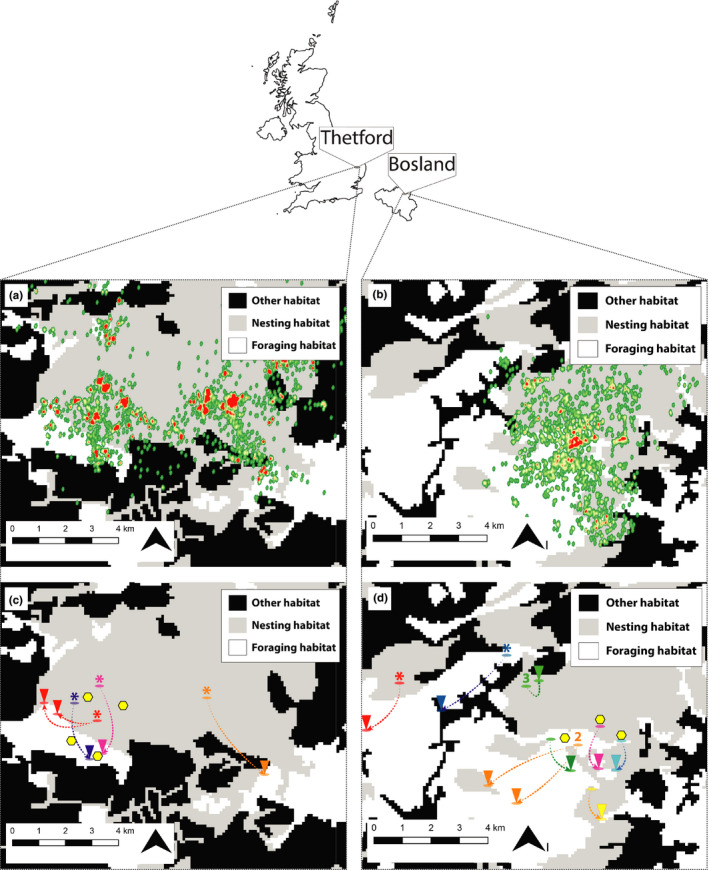
Heat maps of nightjars' functional habitat use (a, b) and precise locations of nesting and foraging sites of sampled individuals (c, d) in Thetford (T; a,c) and Bosland (B; b,d). The functional habitat layer is a simplified Corine habitat layer with nesting habitat (gray; both in forest and heathland) and foraging habitats (white; extensively grazed or—cultivated grasslands) and other habitats (black; for example: urban areas and agricultural land). The heat maps show the distribution of GPS observations (a; 2014–2016) and radio telemetry observations (b; 2010–2014). Red areas indicate high concentrations of observations, both in nesting and foraging areas. Maps c and d are derived from radio telemetry or GPS observations and show the nesting (ovals in gray areas) and foraging locations (ovals with a triangle in white areas) of sampled individuals. One color is one individual, except when numbers are placed above the nesting/foraging location. Numbers represent the amount of individuals that visited a particular site. Locations with an asterisk are based on GPS observations, and all other locations are derived from radio telemetry observations. Yellow hexagons represent locations where food availability was sampled

We collected information on the spatial habitat use of nightjars, spatial variation in food availability, and nightjars' dietary composition in two main study areas in Belgium (Bosland; 51.17°N, 5.34°E) and the United Kingdom (Thetford Forest; 52.45°N, 0.65°E) (Figure 1). In both study areas, the nesting habitats used by nightjars are lowland commercial pine forest on dry, sandy soils (Conway et al., 2019; Evens et al., 2017) and nightjars are presumed to forage away from their nesting sites in complementary habitats (Conway et al., 2019; Evens et al., 2017).

### 2.1 Spatial habitat use

We reanalyzed data on the spatial habitat use of nightjars, collected by radio telemetry (Bosland: 2010–2014; May to August (Evens et al., 2017)) and high‐resolution GPS‐tracking (Bosland: 2014; May–August and Thetford Forest: 2014–2016; June–July (Conway et al., 2019)).

We reclassified the 2018 CORINE Land Cover (European Environment Agency, 2019) into three functional habitat types: nesting, foraging, and other (Evens et al., 2018). Nesting habitats are defined as open habitats within the vicinity of nests, such as heathlands and forest clearings and foraging habitats are open habitats away from the nest sites, such as extensively grazed grasslands, grass‐heath, wet meadows, and arable crops. Other habitats are not used for nesting or feeding, mainly comprising urbanized areas and dense forests.

To show the full use of functional habitats in the same timeframe as when the fecal pellets were collected, we constructed a heatmap (10 m radius; QGIS 3.6.0) containing all observations of all nightjars tracked in Bosland and Thetford: 2,753 radio telemetry observations (48 individuals; Bosland (Evens et al., 2017) and 17,817 GPS positions (29 individuals; Thetford Forest; Conway et al., 2019; Figure 1a,b). To show the specific use of functional habitats of individuals from which fecal samples were collected, we then visualized the exact locations of nesting and foraging sites of those individuals in the year fecal samples were collected (Figure 1c,d).

### 2.2 Food availability

For the purpose of this study, we focussed on moths (*Lepidoptera*) as these were expected to constitute the main diet of nightjars (Sierro et al., 2001). We used average species‐specific wing length (Waring & Townsend, 2017) as a measure to classify macro‐moths into ten size categories (hereafter referred to as size group; Table 1). We defined food availability as the number of individuals per species or family and size group. The use of low‐power light traps allows the collection of local estimates of food availability within the focal area of the nesting or foraging habitat (Merckx & Slade, 2014).

**Table 1:** Moth size group according to forewing size (mm)

Wing (mm)	< 10	10–11	12–13	14–15	16–17	18–19	20–21	22–23	24–25	>25
Size group	1	2	3	4	5	6	7	8	9	10

In Bosland, we quantified the availability of Lepidopterans during four consecutive years (2011–2014, May–August; same period as fecal pellets were collected). We used six 15 Watt UVA‐lamps simultaneously in the two functional habitats simultaneously (two traps per trapping site; attraction radius for photosensitive insects is approximately 5 m; for methodological details see Evens et al. (2018); Figure 1c). Specimens of macro‐moths were removed from the liquid at dawn, dried, and identified to species‐level.

In Thetford Forest, we trapped moths in 2016 (June and August) in the two functional habitats simultaneously and we also used similar low‐power 15‐Watt actinic light (attraction radius for photosensitive insects is approximately five meters). Eight traps were initially used simultaneously (two in each of the two nesting habitats and 4 in the foraging habitat) and operated on the same night; subsequently, two traps were placed in one of the breeding habitats and alternated with the other breeding habitat on subsequent nights, while maintaining 4 traps per night within the foraging habitat (Figure 1c). Moth traps were set at dusk and covered at dawn, after which identifications were performed in the field. Only specimens of macro‐moths were identified to species level but all specimens were recorded.

Inter‐ or intra‐annual variation in prey availability was not considered. We further assume that general moth communities within functional habitats remained the same between years (Habel et al., 2019) considering the lack of structural landscape changes in these habitats during this short‐term sampling period.

### 2.3 Dietary composition from DNA

We collected fecal samples at active nightjar nesting or roosting sites, comprising 20 nests and two roosts in Bosland and seven nests in Thetford Forest. Nests and roosts were located on the ground and in shaded areas under the canopy. The two roosting sites of males were located within breeding habitat and in the immediate proximity (>100 m) of active nests. At nests, we targeted fresh, intact fecal samples of adult birds (±1 cm diameter), which are considerably larger than those of young chicks (±0.5 cm diameter; personal observations). During single sampling opportunities, we collected the fecal samples when (a) nests of tracked adults were inspected for the first time, (b) nests of tracked adults were visited to ring the chicks, (c) upon discovery of new nests (adults were tagged after nest discovery), or (d) when roosts of radio‐tagged adults were found. For each nest or roost, the pellets were dried and stored in small vials. Although fresh samples were targeted, the exact age of the fecal pellets could not be determined. Based on field observations, we expect that most samples were one to four weeks old.

We extracted DNA from each fecal sample following the QIAamp® DNA Stool Mini Kit “Isolation of DNA from Larger Volumes of Stool” protocol (Qiagen) with slight modifications detailed in (Zeale et al., 2011), using negative extraction controls (*n* = 15) throughout. We used universal “mini‐barcode” primers jgLCO1490 (5′‐TNTCNACNAAYCAYAARGAYATTGG‐3′ (Geller et al., 2013), and EPT‐long‐uniRed (5′‐AARAAAATYATAAYAAANGCGTG‐3′; modified from Hajibabaei et al. (2011) to amplify a 133 bp region of the cytochrome c oxidase (COI) mitochondrial gene. These small fragments, of which similar size fragments have successfully been used in other studies (Galan et al., 2018; Gillet et al., 2015), were chosen due to the highly degraded nature of the dietary DNA in the fecal samples. The selection of these primers followed extensive testing of multiple primers pairs across invertebrate taxa (Stockdale, 2018). We also considered other primers suited to surveying Lepidoptera (Zeale et al., 2011), however, as they are known to be subject to massive amplification bias (Piñol et al., 2019) and provide only adequate coverage for other species which are known to comprise the diet of nightjars (e.g., Coleoptera (Clarke et al., 2014)), they were not used. The selection of these primers followed extensive testing of multiple primers pairs across invertebrate taxa (Stockdale, 2018). We labeled each fecal sample with a unique combination of HTS grade forward and reverse 10 bp multiplex identifier tags (MID tags (Brown et al., 2007). We carried out PCRs in 10 µl reaction volumes containing 5 µl multiplex buffer (Qiagen), 1.7 µl H_2_O, 0.2 µl forward primer (10 µM), 1 µl reverse primer (2 µM) added individually, 0.1 µl Bovine Serum Albumin (BSA; New England Biolabs), and 2 µl DNA. Reaction conditions consisted of an initial denaturation at 95°C for 15 min; 35 cycles of 94°C for 30 s, primer‐specific annealing temperature (48°C for 133 bp fragment and 50°C for 306 bp fragment) for 90 s, 72°C for 90 s, and a final extension of 72°C for 10 min.

We visualized the samples under UV light on a 1.5% agarose gel stained with SYBR®Safe (ThermoFisher Scientific) and pooled them according to intensity of the PCR product when compared to a standardized 100 bp ladder. We used a BioAnalyzer (Agilent Technologies) to check the pooled peak amplicon size, determine DNA concentration, and to check for the presence of primer dimer. We purified pooled samples of similar DNA concentration using Agencourt AMPure XP purification beads (Beckman Coulter) and quantified DNA concentrations using a Qubit (ThermoFisher Scientific). We then combined the samples in order to provide a final overall pooled sample with an approximately equal amount of amplicon DNA from each fecal sample. Finally, we used purification beads to perform a final clean‐up of the overall pools of individually tagged amplicons and remove any remaining primer dimer. We used the NEXTFlex Rapid DNA‐seq Library Prep Kit for Illumina (Bioscientific) to prepare the pooled MID tagged amplicons for paired end sequencing. The prepared library was sequenced separately using 150 bp paired‐end reads, on a MiSeq desktop sequencer (Illumina).

To identify invertebrate species, we filtered paired‐end Illumina sequences for quality using Trimmomatic v0.36 (Bolger et al., 2014) with a minimum quality score of 20 over a sliding window of 4 bp, and a minimum length of 80 bp. These were aligned using FLASH (Magoč & Salzberg, 2011) and de‐multiplexed into fecal sample‐specific files using the MID tag sequence with the “trim_seqs” command in Mothur (Schloss et al., 2009), which also removes the MID tag and primer sequences from the reads. We condensed the sequences into molecular operational taxonomic units (MOTUs) using USEARCH software v9.2.64 (Edgar, 2010), first by de‐replicating to remove any infrequent haplotypes with fewer than 10 sequences within a fecal sample, then discarding potential chimeras (“uchime2_denovo”) and finally clustering at 97% similarity.

We assigned the resulting sequences to taxonomic units using the BLAST algorithm (Altschul et al., 1997) to search GenBank, using a cut‐off of 90% sequence identity. If a sequence matched only one species on GenBank with 99% sequence identity, the sequence was assigned to that species. If the sequence matched with 98% sequence identity, the sequence was assigned to genus. Finally, if the sequence matched more than one species from the same genus, tribe, or family, the lowest common taxonomic unit up to the order level was assigned. Any sequence with <90% match to the closest matching species on GenBank, or for which BLAST returned no significant match was discarded, as was any sequence for which the closest match included bacterium, gastrotrich, fungus, or algae (Hawkins et al., 2015; Razgour et al., 2011) and slugs, spiders and worms as they are unlikely to represent prey species (Sierro et al., 2001; Schlegel, 1967).

To clean data prior to statistical analysis, we applied a sequence read‐number approach (Dunn et al., 2018) to remove all read counts less than the maximum present in negative controls and unused MID tag combinations. Finally, we combined multiple sequences matching the same taxonomic unit.

### 2.4 Statistical analysis

We used the R‐package “econullnetr” to model resource selection (Vaughan et al., 2017) by nightjars in Bosland and Thetford. This method determines the significance of interactions between food availability (resource) and food choice (resource use) by mirroring the interaction terms of an observed network (e.g., available and consumed moths of a certain size group) with random resource use (null model; e.g., moths of a certain size group are consumed in proportion to their availability). Although this method cannot explain the mechanisms underpinning resource choice, it can provide a way to highlight interactions between food availability and food choice (Vaughan et al., 2017). To investigate whether nightjars prefer moths of a certain size group, we pooled consumer data (diet composition: number of DNA records per moth size group) and resource data (resource composition: number of moths per size group) per site (Bosland or Thetford). To investigate whether nightjars prefer moth families of a certain size group, we also pooled consumer data (diet composition: number of DNA records per moth family and size group) and resource data (resource composition: number of moths per family and size group) per site (Bosland or Thetford).

## 3 RESULTS

### 3.1 Spatial habitat use

Tracking data from both study sites show that nightjars use complementary habitats to breed and to forage (Figure 1). The exact locations of nesting and foraging sites were identified for eleven (Bosland) and four (Thetford) individuals from which we collected dietary data (Figure 1). Median foraging distances for these individuals were 1.5 km (Bosland, *n* = 9, range = 0.5–3.7 km) and 2.2 km (Thetford, *n* = 5, range = 1.5–3.7 km) (Figure 1). Nesting sites were always located in heathlands, whereas foraging sites comprised extensively grazed grasslands or grass‐heath and meadows.

### 3.2 Food availability

**Figure 2:**
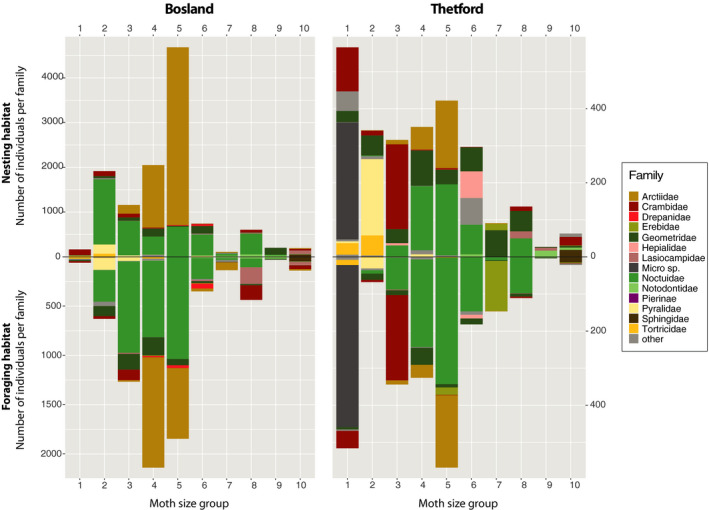
Distribution plots: bar plots indicating the number of Lepidopterans per size group and family found in environmental samples (top = nesting habitat, bottom = foraging habitat) for Bosland (left) and Thetford (right)

Environmental samples were collected during 58 (Bosland) and 20 (Thetford) sampling nights and contained 18,855 (Bosland; 324 species) and 4,899 individuals of *Lepidoptera* (Thetford; 238 species) (Figure 2), mainly consisting of *Arctiidae* (Bosland: 40%, Thetford: 10%), *Crambidae* (Thetford: 14%), *Geometridae* (Bosland: 7%, Thetford: 12%), unidentified micro moths (Thetford: 15%), and *Noctuidae* (Bosland: 41%, Thetford: 30%). Micro moths were not considered in Bosland.

### 3.3 Diet composition from DNA

We successfully amplified DNA from 48 sets of fecal samples (Bosland: 41, Thetford: 7) pooled into a total of 29 different nest/roost locations (Bosland: 22, Thetford: 7). From all the sets of fecal samples, we identified 2,027 unique sequences of which 1,756 sequences were either poor quality sequences or identified as contamination caused by *Fungi*, *Algae, Bacteria, or* invertebrates that were unlikely to represent prey species, such as slugs, spiders, or worms. The other 271 sequences comprised 166 unique molecular taxonomical units representing possible prey species. When combining data from all samples, a total of 418 occurrences (e.g., one taxonomical unit can be recorded in several samples) represented possible prey species. We identified 90% of prey sequences to the species level (84% of taxonomic units) and an additional 10% to the genus level (16% of taxonomic units). Prey items were identified as *Lepidoptera* (65%; Moths), *Diptera* (21%; consisting of Brachycera [Flies, 18%], Tipulidae [Craneflies, 2%] and 1% of Nematocera [Gnats], Syrphidae [Hoverflies] and Culicidae [Mosquitos]), *Coloptera* (10%; Beetles associated to grasslands, pine – and deciduous forests), *Ephemeroptera* (2%; Mayflies) and other less‐represented groups (in total 2%) such as *Trichoptera* (Caddisflies), *Hymenoptera* (Ants), *Dermaptera* (Earwigs), and *Plecoptera* (Stoneflies) (Table 2). Lepidopterans comprised 16 moth families (Bosland: 52 species; Thetford 35 species) and one butterfly family (Appendix S1), mainly represented by *Noctuidae* (Bosland: 49%, Thetford: 53%) and *Geometridae* (Bosland: 17%, Thetford: 11%). Consumed species of *Lepidoptera* were not always detected in environmental samples. In Bosland, 28% of consumed species remained undetected, with 35% undetected in nesting habitat and 35% in foraging habitat. In Thetford, 66% of consumed species remained undetected, with 75% undetected in nesting habitat and 64% in foraging habitat.

**Table 2:** Table summarizing the proportion of insect orders and families found in the DNA samples

Order and family	Percentage
*Lepidoptera* ^a^ (Moths)	65
*Noctuidae*	54.3
*Geometridae*	13.8
*Erebidae*	6.4
*Lasiocampidae*	4.3
*Notodontidae*	4.3
*Drepanidae*	3.2
*Pierinae*	3.2
*Other* ^b^	10^b^
*Diptera* (Two‐winged Flies)	21
*Brachycera, Tipulidae, Nemotocera, Syrphidae, Culicidae*	
*Coloptera* (Beetles)	10
*Chrysomelidae, Mycetophagidae, Cerambycidae, Carabidae, Cantharidae, Leiodidae*	
*Ephemeroptera* (Mayflies)	2
Other	2
*Trichoptera* (Caddisflies)	
*Hymenoptera* (Ants)	
*Dermaptera* (Earwigs)	
*Plecoptera* (Stoneflies)	

Note

Percentages are shown for each order and families within each order.

^a^ 9 out of 17 families are shown, representing approximately 90% of Lepidopterans.

^b^ Crambidae, Hepialidae, Lymantriidae, Sphingidae, Thyatiridae, Tortricidae.

### 3.4 Resource selection

**Figure 3:**
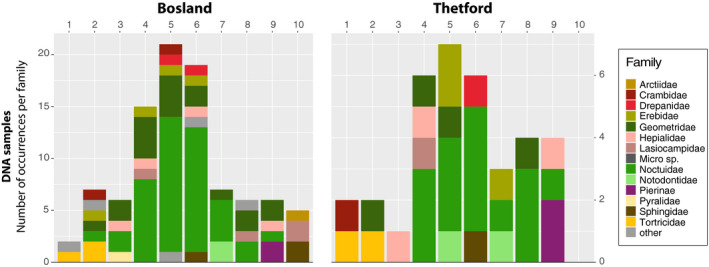
Distribution plots: bar plots indicating the number of occurrences per size group and family found in DNA samples for Bosland (left) and Thetford (right)

Visual inspection of the number of occurrences per moth families in environmental and DNA samples suggests that species of *Lepidoptera* with a forewing size between 10 and 17 mm (size group 2 and 5) were most available in environmental samples, and species with a forewing size of 14‐19 mm (size group 4, 5, and 6) were most detected in the DNA samples (Figure 3).

**Figure 4:**
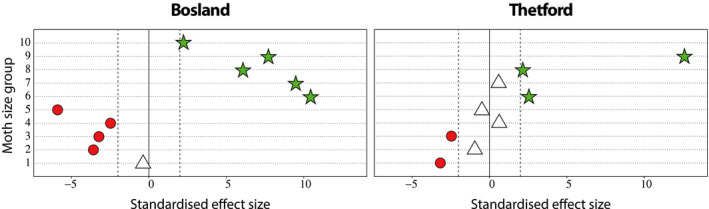
Preference plot for nightjars in Bosland and Thetford, comparing the observed interaction frequencies (dots) to the 95% confidence intervals from the null model (vertical dashed lines). The interaction represents occurrences of moth size groups in environmental samples (resource: available food) and fecal samples (consumed: DNA analysis). The green star denotes an interaction that was stronger than expected under the null model and the red dot weaker than expected. White triangles denote interactions that were not significantly different from the null model

**Figure 5:**
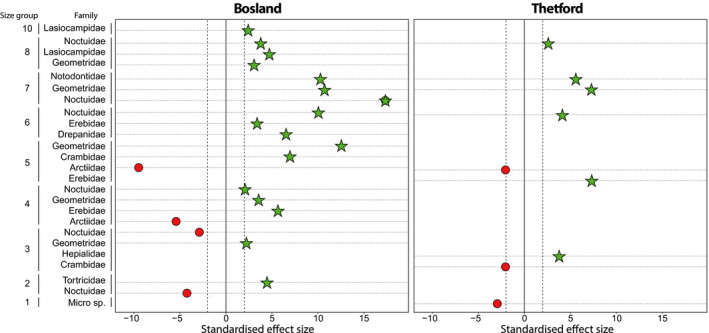
Preference plot for nightjars in Bosland and Thetford, comparing the observed interaction frequencies (dots) to the 95% confidence intervals from the null model (vertical dashed lines). The interaction represents occurrences of moth families per size group in environmental samples (resource: available food) and fecal samples (consumed: DNA analysis). The green star denotes an interaction that was stronger than expected under the null model and the red dot weaker than expected. Moth family‐size groups that did not show significant interactions, or which were not recorded in the environmental samples are not shown

The resource selection models show that nightjars select larger and avoid smaller groups of Lepidopterans. The consumption of larger‐sized moths (forewing size longer than 17 mm; Bosland: size group 6–10, Thetford: size group 6, 8, and 9) is higher than their availability (Figure 4). The consumption of smaller‐sized moths (forewing size shorter than 18 mm; Bosland: size group 2–5, Thetford: size group 1 and 3) is lower than their availability (Figure 4). Similarly, the resource selection models suggest that some species of Lepidopterans are selected, such as *Geometridae*, *Noctuidae*, *Lasiocampidae,* and *Erebeidae* (Figure 5) whereas smaller species of *Arctiidae*, *Noctuidae,* and micro moths (forewing size < 10 mm, size group 1) are avoided (Figure 5).

## 4 DISCUSSION

Our study gives insights into the functional relationship between food availability, spatial use, and diet of a crepuscular aerial insectivore. From fecal samples, using high‐throughput sequencing and DNA‐metabarcoding, we show that species of *Lepidoptera*, *Diptera,* and *Coleoptera* form the main components of nightjars' diet, as expected. Our results further suggest that nightjars select larger species and avoid smaller species of *Lepidoptera*. Other prey, such as species of *Tipulidae*, *Plecoptera,* and *Ephemeroptera*, are typically associated only with foraging habitats (i.e., grasslands and aquatic environments).

### 4.1 Diet composition

Our study provides the first molecular‐based insights into the diet of nightjars, which comprises species of *Lepidoptera*, *Diptera*, *Coloptera*, *Ephemeroptera*, *Trichoptera*, *Hymenoptera*, *Hemiptera*, *Orthoptera*, *Dermaptera,* and *Plecoptera* (Table 2). Assuming similar patterns of habitat selection, food availability, and selection between sites, our findings diverge from earlier, morphology‐based studies because the proportion of identified Dipterans was higher, and the proportion of Coleopterans was lower compared with previous estimates (Sierro et al., 2001; Schlegel, 1967). Furthermore, we did not detect any species of *Neuroptera* or *Odonata* (Sierro et al., 2001; Schlegel, 1967).

In our study, approximately 96% of the prey sequences in all fecal samples comprised Lepidopterans (65%), Coleopterans (10%), and Dipterans (21%) (Table 2). Resource selection models show that nightjars prefer larger‐sized Lepidopterans (forewing > 19 mm; Figure 4). More specifically, larger species of *Geometridae*, *Lasiocampidae,* and *Noctuidae* were consumed more, relative to their availability, whereas smaller species of the same families and micro moths were consumed less (Figures 3 and 5). Furthermore, species of *Arctiidae* were significantly less represented from fecal samples despite their relatively high occurrence in environmental samples. Reasons for the low abundance in the diet currently remain poorly understood, yet many of the *Arctiidae* are generally unpalatable to birds (Rojas et al., 2017). Alternatively, the ecological differences between species of Lepidopterans might contribute to their presence or absence in nightjars' diet. Nightjars typically hawk prey from below in steep flight while the prey is silhouetted against the bright sky (Alexander & Cresswell, 1990; Camacho, 2014; Evens et al., 2018; Jackson, 2003). As such, activity patterns, foraging ecology or flight behavior of moths (Merckx et al., 2012), or different attractions to light (Merckx & Slade, 2014) are probably influencing the interactions between nightjars and their prey (English et al., 2018).

Although it is widely recognized that Coleopterans are a common food item for nightjars, Lepidopterans are known to be more energy rich in comparison (Bayne & Brigham, 1995). The number of Coleopterans found in our study is low when compared with traditional previous morphology‐based studies (38% (Cramp et al., 1985); 20% (Schlegel, 1967); 5%–18% (Sierro et al., 2001), but similar (2% of fecal pellets) to that found in Thetford Forest (Sharps et al., 2015). It has been suggested that young chicks are mainly fed with soft‐bodied insects (Sierro et al., 2001). Yet, all the investigated fecal samples in our study have been collected from adult nightjars (at nest sites); and thus, a potential sampling bias unlikely explains the lack of Coleopterans in the diet of adult nightjars. Also, Coleopterans are more difficult to digest when compared with soft‐bodied insects such as moths and flies (Garlapow, 2007). We can expect that morphology‐based assessments of fecal pellets are potentially biased toward the identification of undigested chitinous exoskeletons. We do believe, however, that Coleopterans occasionally can be the predominant food source for nightjars (visual inspection of food pellets), for example during outbreaks of *Cerambycidae* emerging from cut pine wood stumps (Hedgren, 2007). Finally, as with many other invertebrate groups, there have been long‐term population declines among the majority of Coleopterans within North‐Western Europe (Brooks et al., 2012). This may indicate that the small component of Coleopterans in nightjar diet reflects a dietary shift in response to a wider reduction in the availability of this prey type, although more research is required to support this idea.

Concerning the Dipterans, it is noteworthy that more than 20% of prey items were Dipterans from different families. Species of *Tipulidae* and *Muscidae* are not surprising as a prey source, since they occur in known foraging habitats such as grasslands, arable lands, and wet or aquatic environments. The detected species of *Brachycera* are mainly pollinators, parasites, fungivores, or detritivores and can also occur in high numbers at night during certain periods of the breeding season (personal observations).

Neuropterans and Odonates were not found as prey in our study, and probably comprise marginal fractions of nightjars' diet (Sierro et al., 2001; Schlegel, 1967). Neuropterans, such as species of *Chrysopidae*, have been observed in food pellets of nightjars during our study (personal observations in food pellets). We also expect that Odonates are only occasionally eaten by nightjars at dusk or dawn since Odonates are predominantly diurnal insects (Corbet, 1999).

Our findings suggest that the molecular identification of invertebrate prey from fecal samples provides comprehensive information on the diet of nightjars. This approach is more straightforward than traditional, time consuming morphology‐based methods and also avoids potential observer biases and the application of invasive sampling techniques, such as the use of neck collars (Sierro et al., 2001). The applied methods provide a semi‐quantitative measure of prey consumption (Bowser, Diamond, & Addison, 2013; Deagle, Thomas, Shaffer, Trites, & Jarman, 2013) and that comparisons between prey availability (i.e., abundance data) and consumption (i.e., presence/absence data) should be made carefully because large numbers of feces are generally required to reliably detect a wide array of consumed prey species in field‐collected samples (Thalinger et al., 2017). In this study, we pooled approximately 250 fecal samples (into 48 sets of fecal samples related to nests or roosts) and we detected 166 unique taxonomical units, comprising 418 possible prey items. The wide range of invertebrate prey detected in fecal samples of nightjars shows that molecular approaches are a versatile tool to examine birds' diet. However, among other things, sample conditions, contamination (Mcinnes et al., 2016; Oehm et al., 2011), and meal size (Gagnon et al., 2011; Juen & Traugott, 2005) can complicate the interpretation of DNA‐based data obtained from field‐collected feces (Thalinger et al., 2017). Well‐stored fecal samples should be useful for metabarcoding (Rytkönen et al., 2019), yet amplification success and proportion of food DNA persisting in samples reduce when exposed to sunlight, rain, or even the forest floor (Mcinnes et al., 2016; Oehm et al., 2011). Dried fecal samples also have had more potential exposure to external contamination, from fungi and other invertebrates (Oehm et al., 2011), which may explain the presence of non‐food DNA sequences in our study. We cannot be excluded the fact that prey DNA was lost from our samples albeit (a) we targeted fresh, intact feces, during a single sampling opportunity; (b) nests and roosts were usually in shaded areas under the canopy which may allow amplifiable DNA to persist for longer (Mcinnes et al., 2016); (c) and we used a shorter universal primer pair to account for the degraded DNA.

### 4.2 Spatial use and food availability

The diet analyses indicate a broad range of prey items, including species of *Lepidoptera*, *Tipulidae*, *Plecoptera,* and *Ephemeroptera,* which are associated with terrestrial and aquatic environments in foraging habitats. The main habitat preference of approximately 47% species of Lepidopterans could also not be directly associated with nightjars' nesting habitat (i.e., heathlands, coniferous forests, and deciduous forests; habitat preferences derived from www.vlinderstichting.nl). Although we should interpret the Thetford data with caution, since only a small number of fecal samples was used for DNA‐metabarcoding, overall the observations from our study seem representative as they are in accordance with previous studies and the sampled nightjars' spatial use (Figure 1). Similar to earlier studies in the same research areas, nightjars commute between semi‐open heathlands (nesting habitat) and distant grasslands, swamps, meadows close to streams, and grazed‐grassland heath (Conway et al., 2019; Evens et al., 2017, 2018). These studies indicate that in Bosland higher moth biomass in foraging habitats might be the reason for this regular commuting behavior at dusk and dawn (Evens et al., 2018). In Thetford, no such differences in moth biomass were identified (Sharps et al., 2015). Following these results, we believe there are two factors promoting the observed foraging behavior. Firstly, high habitat diversity and structural and micro‐climatic heterogeneity, found in foraging habitats, are important for maintaining the diversity and density of Lepidopterans (Sánchez‐Bayo & Wyckhuys, 2019) by larval food plant availability, nectar sources for adults, presence of landmark features for mating and dispersal and shelter (Dover & Settele, 2009; Pywell et al., 2004). Secondly, nightjars are visual hunters which hawk prey from below in steep flight while the prey is silhouetted against the bright sky (Alexander & Cresswell, 1990; Camacho, 2014; Evens et al., 2018; Jackson, 2003). Available light (during twilight or moonlit nights) is an important factor limiting optimal foraging conditions (Evens et al., 2020), which potentially are compromised within shaded forests (Sharps et al., 2015), for example, compared to more open foraging destinations. Alternatively, larger prey may be easier to detect in darker conditions. Following the predictability of high food resources and light conditions, optimal foraging theory predicts that diet choice and foraging behavior should select the most energetically favorable prey items (Stephens & Krebs, 1985). It means that high prey densities and the selection of larger prey may allow for increased gross food intake by a generalist, such as the nightjar.

## 5 CONCLUSION

We used high‐throughput sequencing and DNA‐metabarcoding to investigate the diet of European Nightjars in relation to food availability and the birds' spatial use. Nightjars are generalist insectivores, with a preference for larger Lepidopterans from which a substantial part of their daily nourishment is probably collected in foraging sites. To increase gross food intake during such foraging trips, nightjars seem to select the most energetically favorable—larger—prey items in habitats containing the highest prey densities (Evens et al., 2018). These outcomes confirm earlier assumptions (Alexander & Cresswell, 1990; Evens et al., 2017, 2018) that a considerable proportion of nightjars' daily diet originates from habitats which were, until recently, presumed to be unsuitable for this crepuscular bird species. These findings, again, highlight the importance of dietary studies for the implementation of effective species‐based conservation strategies (Catry et al., 2019).

Across Europe, long‐term monitoring of moth abundance since the 1960s has shown that around a third of common moth species are in decline (Fox et al., 2013; van Langevelde et al., 2018; Saunders et al., 2020). Especially populations of larger‐winged moths seem to decline more significantly compared to shorter‐winged species (Coulthard et al., 2019; Potocký et al., 2018), except in the case of urbanized environments (Merckx et al., 2018). As nightjars are predominantly reliant upon larger moths, this raises questions about how changes in invertebrate communities may affect their behavior and space use, and how this will impact their distribution and abundance.

## CONFLICT OF INTEREST

We declare we have no competing interests.

## AUTHOR CONTRIBUTIONS

**Ruben Evens:** Conceptualization (lead); data curation (lead); formal analysis (equal); funding acquisition (equal); investigation (equal); methodology (equal); supervision (lead); validation (equal); visualization (lead); writing–original draft (lead); Writing–review and editing (lead). **Greg J. Conway:** Conceptualization (lead); data curation (equal); formal analysis (equal); funding acquisition (equal); investigation (lead); methodology (equal); supervision (lead); validation (lead); visualization (equal); writing–original draft (lead); writing–review and editing (equal). **Kirsty Franklin:** Data curation (equal); formal analysis (lead); methodology (lead); validation (equal); writing–review and editing (equal). **Ian G. Henderson:** Writing–review and editing (equal). **Jennifer Stockdale:** Funding acquisition (equal); investigation (equal); methodology (equal); validation (equal); writing–review and editing (equal). **Natalie Beenaerts:** Validation (equal); writing–review and editing (equal). **Karen Smeets:** Funding acquisition (lead); writing–review and editing (equal). **Thomas Neyens:** Formal analysis (equal); methodology (lead); validation (equal); writing–review and editing (equal). **Eddy Ulenaers:** Data curation (equal); investigation (equal); writing–review and editing (equal). **Tom Artois:** Funding acquisition (equal); Supervision (equal); validation (equal); writing–review and editing (equal).

### Open Research Badges

This article has earned an Open Data Badge for making publicly available the digitally‐shareable data necessary to reproduce the reported results. The data is available at https://osf.io/3dytb/.

## Supporting information

Appendix S1

## Data Availability

Food availability datasets and occurrences of invertebrate species in fecal samples are available from the OSF‐repository: https://osf.io/3dytb/
