# Supplementary material for: DNA diet profiles with high‐resolution animal tracking data reveal levels of prey selection relative to habitat choice in a crepuscular insectivorous bird
Source: Ecol Evol. 2020 Oct 16;10(23):13044–56. doi: 10.1002/ece3.6893 (PMC7713983; doi:10.1002/ece3.6893)
Supplement: Supplementary file 1 — Appendix S1 [file ECE3-10-13044-s001.docx]

**Appendix S1: Proportion of Lepidopterans**

*Table A1: Proportion of families of Lepidopterans in DNA and food availability samples in Bosland (B) and Thetford Forest (T).*

| Family | DNA Total (%) | DNA B (%) | DNA T (%) | Food B (%) | Food T (%) |
| --- | --- | --- | --- | --- | --- |
| *Arctiidae* | - | - | - | 40.2 | 10.2 |
| *Cossidae* | - | - | - | < 0.1 | < 0.1 |
| *Crambidae* | 2.1 | 1.7 | 2.9 | 4.1 | 14.1 |
| *Depressariidae* | - | 0 | - | - | - |
| *Drepanidae* | 3.2 | 3.4 | 2.9 | 0.8 | < 0.1 |
| *Erebidae* | 6.4 | 3.4 | 11.4 | 0.1 | 3.6 |
| *Geometridae* | 13.8 | 15.3 | 11.4 | 6.6 | 11.8 |
| *Glyphipterigidae* | - | - | - | < 0.1 | - |
| *Hepialidae* | 2.1 | 1.7 | 2.9 | < 0.1 | 1.8 |
| *Lasiocampidae* | 4.3 | 5.1 | 2.9 | 1.8 | 0.6 |
| *Limacodidae* | - | - | - | 0.4 | - |
| *Lymantriidae* | 1.1 | 1.7 | - | 0.4 | 1.7 |
| *Noctuidae* | 54.3 | 57.6 | 48.6 | 40.7 | 29.6 |
| *Nolidae* | - | - | - | 0.1 | < 0.1 |
| *Notodontidae* | 4.3 | 3.4 | 5.7 | 0.9 | 0.7 |
| *Nymphalidae* | - | - | - | < 0.1 | - |
| *Oecophoridae* | - | - | - | 0.1 | 0.2 |
| *Pierinae* | 3.2 | 1.7 | 5.7 | < 0.1 | - |
| *Plutellidae* | - | - | - | < 0.1 | - |
| *Pyralidae* | - | - | - | 2.2 | 5.1 |
| *Sphingidae* | 2.1 | 3.4 | - | 0.5 | 0.7 |
| *Thaumetopoeidae* | - | - | - | 0.3 | - |
| *Thyatiridae* | 1.1 | 1.7 | - | - | < 0.1 |
| *Tineidae* | - | - | - | < 0.1 | < 0.1 |
| *Tortricidae* | 2.1 | - | 5.7 | 0.8 | 2.2 |
| *Yponomeutidae* | - | - | - | 0.1 | - |
| *Ypsolophidae* | - | - | - | < 0.1 | 0.4 |
| *Autostichidae* | - | - | - | - | 0.2 |
| *Gelechiidae* | - | - | - | - | 1.2 |
| *Gracillariidae* | - | - | - | - | < 0.1 |
| *Lycophotia* | - | - | - | - | < 0.1 |
| *Macro sp* | - | - | - | - | < 0.1 |
| *Micro sp* | - | - | - | - | 15.4 |
| *Momphidae* | - | - | - | - | < 0.1 |
| *Phycitodes* | - | - | - | - | < 0.1 |
| *Pterophoridae* | - | - | - | - | 0.5 |
